# Statistical mechanics of bone damage: a constitutive model

**DOI:** 10.1007/s00249-025-01749-9

**Published:** 2025-05-03

**Authors:** S. García-Vilana, D. Sánchez-Molina

**Affiliations:** https://ror.org/03mb6wj31 grid.6835.8 0000 0004 1937 028X UPC, EEBE-GIES, Eduard Maristany, 14, 08019 Barcelona, Spain

**Keywords:** Human rib, Cortical bone, Statistical mechanics, Entropy, Constitutive, Damage model

## Abstract

After the elastic regime is surpassed, cortical bone exhibits significant microcracking in its post-elastic mechanical behavior. This work develops a thermodynamically consistent, nonlinear constitutive model based on statistical mechanics, designed to predict the stress–strain relationship and the progression of inter-osteon microcracking. To assess the model’s sufficiency, precise tensile and bending tests were performed in comparison to empirical curves that illustrated theoretical predictions of constitutive relationships. Moreover, entropy increases were quantitatively assessed using model parameters refined through experimental data. A large-size sample was utilized, comprising 51 dog-bone-shaped cortical bone specimens from the 4th ribs of various subjects for uniaxial tensile tests, and 15 complete fourth ribs for bending tests. Displacement and strain fields were meticulously recorded using digital image correlation and video analysis. The model demonstrated robustness, accurately fitting the data from all experimental specimens and revealing correlations between constitutive parameters and anthropometric variables. Entropy calculations provide insights into the behavior of the bone under varying strains: microcracking is minimal at low strains with stress nearly proportional to strain, escalating significantly beyond a critical threshold, thus challenging the linear relationship between stress and strain.

## Introduction

Recent advances in microscopic techniques and extensive experimental data on bone tissue mechanics have driven the development of many constitutive material models. These models range from simple heuristic equations to theoretical approaches based on cortical bone’s microstructure, as observed using scanning electron microscopy (SEM) (Atsumi et al. 2017; Pahr and Reisinger 2020; Kraiem et al. 2020). While heuristic models offer simplicity and ease of use in computational biomechanics (Werner et al. 2019; Stipsitz et al. 2019; Abdi et al. 2023; Ptak et al. 2023), there is growing interest in models that more deeply incorporate the mechanobiology of bone and its microstructure (Schwiedrzik et al. 2013; Zysset and Wolfram 2017; Sabet et al. 2018; Sánchez-Molina and García-Vilana 2024). This includes approaches based on microcontinuum mechanics or models that leverage microstructural parameters (Goda et al. 2012; Panyasantisuk et al. 2015; Goda and Ganghoffer 2018; Louna et al. 2019). Most of these models assume an initial elastic behavior, which is then extended to account for inelastic effects. For instance, several works include continuous damage (Krajcinovic et al. 1987; Fondrk et al. 1999; Ramtani and Zidi 2001), microdamage accumulation (Luo et al. 2019), and anisotropy coupled with inelasticity (Atsumi et al. 2017; Iwamoto et al. 2005; Ott et al. 2010). Plasticity and viscoelasticity are also commonly addressed (Natali et al. 2008; Garcia et al. 2009; Luo et al. 2010; Lovrenić-Jugović et al. 2011; Halldin et al. 2014; Lei et al. 2020; Kommidi et al. 2024). Irreversible deformations are typically modeled using continuum damage mechanics or plasticity frameworks. However, effects related to strain rate and thermodynamic considerations—such as entropy production—are often overlooked.

Thermodynamic irreversibility is crucial for understanding how microcracking leads to macroscopic bone fractures. Yet, only a few studies have delved into the thermodynamic aspects of this process (Louna et al. 2018, 2019; García-Vilana and Sánchez-Molina 2023). Only a handful of models explicitly compute the entropy associated with microcracking, a critical factor given its irreversible nature.

This study refines and extends a previously proposed model for predicting the progression of microcracking in bone tissue (García-Vilana and Sánchez-Molina 2023), with a particular emphasis on thermodynamic consistency. Building upon our earlier work, the present model introduces a significant advancement: it is the first to describe the evolution of damage in bone as a natural consequence of the second law of thermodynamics within a statistical mechanics framework. Specifically, the model demonstrates that damage accumulation is intrinsically linked to entropy production, thereby offering a thermodynamically consistent representation of irreversible processes in bone. In addition to enabling the calculation of entropy increases during microcracking, the model is calibrated using experimental data from tensile and bending tests on human rib cortical bone. These experiments allow us to identify correlations between model parameters, anthropometric variables, and the entropy changes associated with microdamage.

## Theory, methods, and data

This section details the theoretical underpinnings of the constitutive material model and describes the experimental calibration of the model’s applicability. It explains the derivation and computation of the entropy increase as well as the stress–strain relationship integral to the model.

### Statistical mechanics of microcracking

**Fig. 1:**
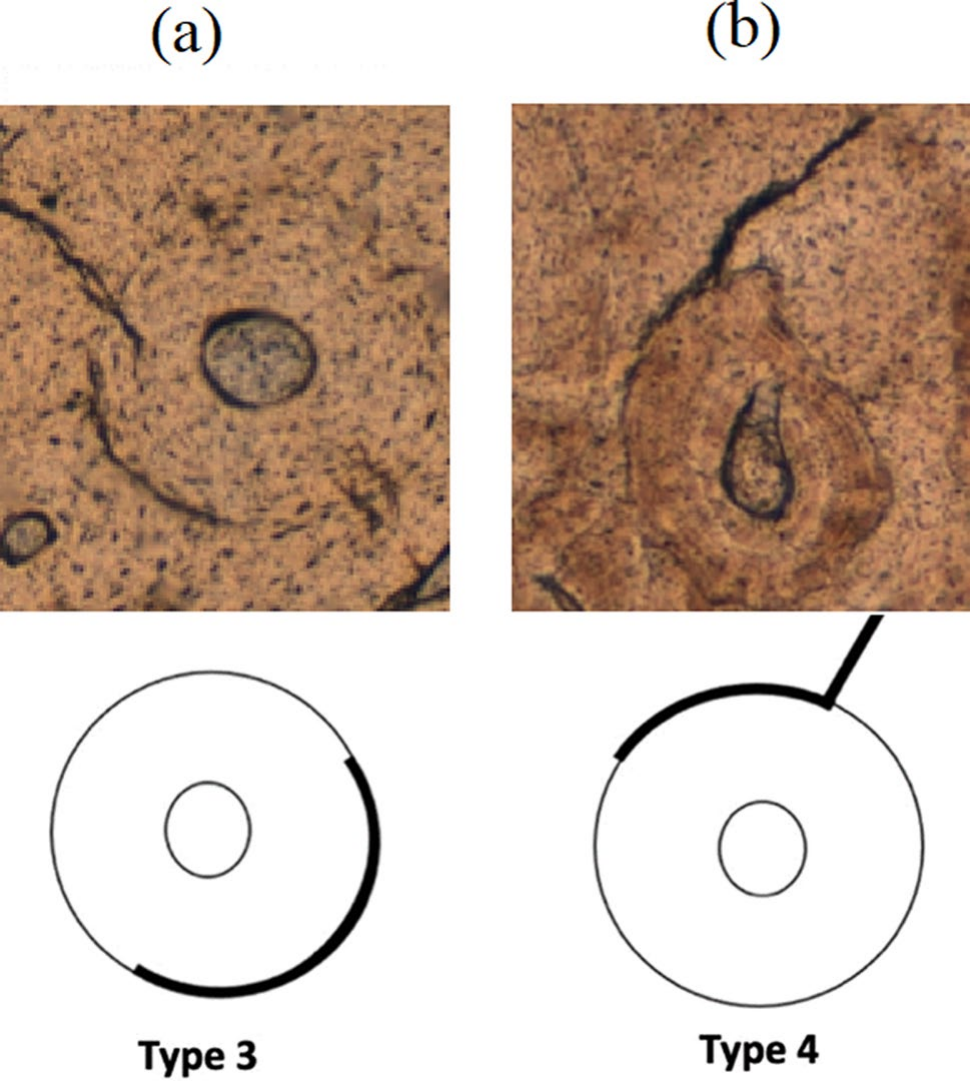
Two types of microcracks circumventing a singular osteon (classification used in Sexton et al. 2024): **a** type 3 and **b** type 4

Cortical bone exhibits a linear mechanical response at small strains, where stress components are proportional to strain components. Experimental observations indicate that intact cortical bone inherently contains microcracks, leading to stress concentrations. As strain increases, the *total microcracking length* (TML), which represents the cumulative length of all microcracks, also increases. Since the inter-osteon space is weaker than the osteons themselves, most microcracks are found in these regions (Zioupos et al. 2008; Wang et al. 2017; Gustafsson et al. 2019; Schwab et al. 2023). For the proposed model, we assume that the majority of microcracks contributing to TML are of types 3 and 4 (see Fig. 1) as classified in Sexton et al. (2024).

Our previous investigations using statistical mechanics (SM) effectively described the interactions between microcracks and osteons, enabling the development of constitutive relationships that accurately replicate the observed stress–strain behavior in cortical bone (García-Vilana and Sánchez-Molina 2023). The current approach is based on two key concepts: (1) cortical bone is treated as an aggregate of osteons that share the applied loads, and (2) when stresses become excessive, microcracks initiate and propagate in the inter-osteonal spaces. Given the random distribution of osteons, SM provides a reasonable framework for modeling the overall mechanical behavior. Since osteons predominantly align along the longitudinal direction of long bones, a transverse-isotropic mechanical behavior is observed (Pezowicz and Glowacki 2012; Mirzaali et al. 2016; García-Vilana et al. 2021). Therefore, we propose a constitutive material model based on five strain invariants (Zheng 1993; Holzapfel 2000).

In subsequent subsections, a transversely isotropic constitutive model is introduced, accounting for both material symmetry and microstructural damage in bone. The SM framework is used to derive a *Strain-Damage Energy Density Function* (SDEDF). Specifically, the *canonical ensemble formalism* of SM allows the inclusion of a *damage variable* ($\alpha$) and a *quenched-disorder parameter* ($\beta$) into the *partition function* $Z(\alpha , \beta ,\varvec{\varepsilon })$ (Dean et al. 2005; Räisänen et al. 1998; Radzihovsky 2015), which is associated with the SDEDF. The quenched-disorder parameter characterizes the initial microcracking in the specimen before stress application, while the damage variable monotonically increases and is linked to entropy increase.

Quenched disorder describes a type of microstructural randomness that is fixed within the material. Unlike other dynamic fluctuations (referred to as annealed disorder), this disorder is intrinsic to the bone’s microstructure and does not evolve over time. The following are key features of quenched disorder:

1. QD remains constant over the time scales of the physical processes under study. Examples of QD in other systems include defects in crystal lattices, grain boundaries in polycrystals, and spatial heterogeneities in disordered media.
2. QD is an intrinsic property of the material and can vary between specimens. This is in contrast to annealed disorder, which averages out over time.
3. QD is modeled statistically, using random variables to describe its impact on stiffness or yield strength at different points within the material.
4. QD can significantly influence macroscopic behavior, including mechanical strength, fracture patterns, transport properties (e.g., heat or electricity), and phase transitions.

In brittle materials such as bone, QD in properties like local toughness or porosity governs crack propagation paths and failure modes. It introduces spatial variability in how the material absorbs and dissipates energy during fracture. Furthermore, microcracking analysis involves combinatorial methods to evaluate the number of possible crack paths along the osteon lattice. The derivation and specific aspects of the proposed model are discussed in detail in the following sections.

#### Partition function and strain-damage energy

Osteons are the fundamental structural units of cortical bone tissue. Together, they form a statistical ensemble that can be analyzed using the SM formalism, as described in detail in García-Vilana and Sánchez-Molina (2023). This formalism enables the quantification of macroscopic quantities associated with a statistical macrostate—in our case, total strain and the total microcracking length (TML) define the macroscopic state. Within the SM framework, the most probable macrostate corresponds to the one with the largest number of compatible microstates (Toussaint and Pride 2002; Shenker 2017; Wallace 2020). Therefore, in the canonical ensemble formalism, it is essential to determine the number of microstates that are consistent with specific macroscopic variables: strain ($\varvec{\varepsilon }$), the damage variable ($\alpha$), and the current level of cracking (TML).

The TML value is obtained by counting the intersections of microcracks with a plane passing through the barycentric axis of the rib bone, as previously detailed (García-Vilana and Sánchez-Molina 2023). This TML value, denoted as $\ell$, is then normalized by the average osteon length $\ell _0$. The resulting *cracking number*, defined as $k = \ell /\ell _0$, represents the number of steps a microcrack has propagated. For a given cracking number (*k*), various possible paths or configurations exist for each microcrack within the inter-osteon lattice. Each path configuration is referred to as a microstate. Therefore, the cracking number (*k*), the strain tensor ($\varvec{\varepsilon }$), and the damage variable ($\alpha$) collectively determine the macrostate energy $E_k(\alpha ,\varvec{\varepsilon })$. The partition function $Z(\alpha ,\beta ,\varvec{\varepsilon })$ is then computed as the following sum (García-Vilana and Sánchez-Molina 2023; Shenker 2017; Wallace 2020):

1 $$\begin{aligned} Z(\alpha ,\beta ,\varvec{\varepsilon })=\sum _{k} g(E_k)e^{-\beta E_k (\alpha ,\varvec{\varepsilon })}. \end{aligned}$$

The number of microstates $g(E_k)$ compatible with such a macrostate and the form of the energy $E_k (\alpha ,\varvec{\varepsilon })$ are given in “Micromechanics of bone and main assumptions”. The relationship between the free Helmholtz energy density ($\Psi$) and the partition function is given by Pathria and Beale (2021)

2 $$\begin{aligned} \Psi (\alpha ,\beta ,\varvec{\varepsilon }) = -\frac{1}{\beta }\ln Z(\alpha ,\beta ,\varvec{\varepsilon }). \end{aligned}$$

This function coincides with the SDEDF, and from it, all the necessary constitutive relations can be derived. Stress tensor ($\varvec{\sigma }$) and entropy (*H*) are given by the following equations:

3 $$\begin{aligned} \varvec{\sigma }(\alpha ,\varvec{\varepsilon }) =\frac{\partial \Psi }{\partial \varvec{\varepsilon }}, \qquad H(\alpha ,\varvec{\varepsilon }) = \beta ^2 \frac{\partial \Psi }{\partial \beta }. \end{aligned}$$

As is well known, the entropy of a system is linked to irreversible processes such as microcracking. Therefore, increases in microcracking can be quantified through corresponding increases in entropy. Entropy plays a crucial role in ensuring thermodynamic consistency. Specifically, Eq. (15) correctly predicts that entropy increases with the level of damage and microcracking. If this condition were not satisfied, the entire statistical mechanics (SM) framework would be invalidated.

#### Micromechanics of bone and main assumptions

As the load increases, the total microcracking length (TML, $\ell = k\ell _0$) and the damage variable ($\alpha$) also increase once the strain surpasses a previously unreached threshold. We assume that the energy of each macrostate in Eq. (1) is given by $E_k=k(1-\alpha ) \psi _0(\varvec{\varepsilon })$ where *k* is the cracking number and $\psi _0$ is a strain energy density function (SEDF), representing the behavior of a transversely isotropic material without cracking. This assumption is founded upon the subsequent observations:

(a) For $k=1$ and $\alpha =0$, the SEDF for an undamaged material is given as $E_k \approx \psi _0(\varvec{\varepsilon })$. (b) The energy associated with cracks is proportional to the total microcracking length (TML, $\ell$), which is expressed as $k = \ell /\ell _0$, as shown in García-Vilana and Sánchez-Molina (2023). (c) Cracking introduces a damage-dependent reduction in stiffness, and since strain energy is proportional to stiffness, the factor $(1-\alpha )$ appears naturally. For instance, in an elastic small rod, the strain energy decreases from $\psi _0 \approx Y\varepsilon ^2/2$ for $\alpha = 0$ to $\psi _0 \approx Y(1-\alpha )\varepsilon ^2/2$ for $\alpha> 0$, where *Y* is the Young’s modulus and $\varepsilon$ denotes the first eigenstrain. (d) Due to the predominant alignment of osteons along the longitudinal axis of long bones, such as human ribs, cortical bone exhibits transversely isotropic behavior. In the cross-sectional plane, the microstructural organization—particularly the osteonal distribution—is approximately uniform in the radial and circumferential directions, supporting the assumption of isotropy within that plane.

Furthermore, the general form of $\psi _0$ for a transversely isotropic material can be expressed in terms of strain invariants, as demonstrated in Zheng (1993), Zheng (1994)

4 $$\begin{aligned} \psi _0(\varvec{\varepsilon }) = \tilde{\psi }_0(I_1(\varvec{\varepsilon }), I_2(\varvec{\varepsilon }), I_3(\varvec{\varepsilon }); I_4(\varvec{\varepsilon },\varvec{a}), I_5(\varvec{\varepsilon },\varvec{a})), \end{aligned}$$

where $\varvec{a}$ is a vector field giving the preferential alignment direction of the osteons along the rib, and the invariants $I_i$ are the trace or *linear invariant* ($I_1$), the *quadratic invariant* ($I_2$), the *determinant* ($I_3$), and the linear and quadratic invariants representing transversal/longitudinal anisotropy ($I_4,I_5$):

5 $$\begin{aligned} \begin{array}{rl} I_1(\varvec{\varepsilon }) & = \text {tr}(\varvec{\varepsilon })\\[1ex] I_2(\varvec{\varepsilon }) & = \frac{1}{2}[\text {tr}^2(\varvec{\varepsilon })-\text {tr}(\varvec{\varepsilon }^2)],\\[1ex] I_3(\varvec{\varepsilon }) & = \det (\varvec{\varepsilon }) \end{array}\quad \begin{array}{rl} I_4(\varvec{\varepsilon },\varvec{a}) & = \varvec{a}\cdot \varvec{\varepsilon }(\varvec{a})\\[1ex] I_5(\varvec{\varepsilon },\varvec{a}) & = \varvec{a}\cdot \varvec{\varepsilon }^2(\varvec{a}). \end{array} \end{aligned}$$

For low-strain conditions where no prior damage has accumulated ($\alpha \approx 0$), cortical bone tissue exhibits a linear mechanical response. Mathematically, these conditions can be represented using a quadratic polynomial in strain as the functional form of $\psi _0$. For example

6 $$\begin{aligned} \psi _0 = P_2(\varvec{\varepsilon }) = \mu _2I_1^2 +\nu _2I_2 + \rho _2I_4^2 +\tau _2I_5. \end{aligned}$$

Once the behavior of cortical bone under low strain has been established, it becomes essential to investigate how damage and strain coevolve as strain increases. Our previous research (García-Vilana and Sánchez-Molina 2023) demonstrated that a third-degree polynomial in strain invariants accurately represents experimental data up to the point just before bone fracture. Accordingly, we propose a relationship between damage and strain that aligns with the context of a monotonic loading process. Specifically, the equation $(1-\alpha )\psi _0 = P_3$, where $P_3(\varvec{\varepsilon })$ is a cubic polynomial of strain invariants, effectively characterizes this relationship.

Under low-strain conditions, this relationship exhibits an approximately linear behavior. However, as strain surpasses a certain threshold, the damage intensifies, necessitating the inclusion of third-order strain invariants. For all these reasons, as well as the observations at the beginning of this section, it is reasonable to propose the following form:

7 $$\begin{aligned} \begin{array}{rl} E_k & = k P_2(\varvec{\varepsilon }) (1-\alpha ) = k P_3(\varvec{\varepsilon })\\ & = k\left( \mu _2I_1^2+\mu _3I_1^3+\nu _2I_2+\nu _3I_1I_2+\varsigma _3I_3+\rho _2I_4^2+\rho _3I_4^3+\tau _2I_5+\tau _3I_4I_5\right) , \end{array} \end{aligned}$$

where $\mu _i, \nu _i, \rho _i, \tau _i$ are parameters of the model that must be fitted. Parameters with a subscript 2 ($\square _2$) represent quadratic functions of the strain components, while those with a subscript 3 ($\square _3$) represent cubic functions of the strain. Terms with degree higher than three in strain have not been considered due to the limited low values of strain in bone.

Equation (7) entails that damage progresses linearly with deformation. Given the relatively small strain that cortical bone undergoes before fracture, this linear assumption is deemed adequate. In practice, based on Eqs. (6) and (7), damage $\tilde{\alpha }$ under monotonic loading conditions is expected to evolve linearly

8 $$\begin{aligned} \tilde{\alpha }(\varvec{\varepsilon }) = -\frac{ \mu _3I_1^3 + \nu _3I_1I_2+\varsigma _3I_3 +\rho _3I_4^3+\tau _3I_4I_5}{\mu _2I_1^2 +\nu _2I_2 + \rho _2I_4^2 +\tau _2I_5}. \end{aligned}$$

Under monotonic loading, the latter form is suitable for reproducing the experimental data, as demonstrated in “Experimental suitability”. However, for more complex loading processes, a more general evolution equation is required, expressed as $\dot{\alpha } = f(\alpha ,\varvec{\varepsilon })$ (Pahr and Reisinger 2020; Holzapfel 2000; Zysset and Curnier 1996), where $\dot{\alpha }$ denotes the time derivative of the damage variable.

In continuum damage models, it is reasonable to assume that damage increases only when an equivalent stress or strain exceeds its previously attained maximum value (Pelà et al. 2011). Typically, this threshold is defined by the maximum value of the corresponding equivalent stress or strain. Specifically, we adopt the following damage progression:

9 $$\begin{aligned} \frac{\text {d}\alpha }{\text {d}t} = {\left\{ \begin{array}{ll} \frac{\text {d}\tilde{\alpha }}{\text {d}\varvec{\varepsilon }}:\dot{\varvec{\varepsilon }}, & \bar{\varepsilon }_t \ge \varepsilon ^+_t \ \text {and}\ \dot{\bar{\varepsilon }}_t>0\\ 0, & \bar{\varepsilon }_t < \varepsilon ^+_t\ \text {or}\ \dot{\bar{\varepsilon }}_t\le 0 \end{array}\right. }, \end{aligned}$$

where $\tilde{\alpha }$ is given by formula (8). With regard to definitory conditions, $\bar{\varepsilon }_t$ is the current value of “equivalent strain”, which is determined from the largest principal strain (eigenstrain) $\varepsilon _I$, as $\bar{\varepsilon }_t = \langle \varepsilon _I(t) \rangle _+$ (where $\langle \cdot \rangle _+$ are the Macaulay brackets which return the value of the argument if positive, return zero otherwise); on the other hand, $\varepsilon ^+_t:= \varepsilon ^+(\varvec{n}_t)$ depends on the direction in which the maximum principal strain is obtained at the instant *t*. The function $\varepsilon ^+(\cdot )$ is evaluated for every fixed direction in space, by means of

10 $$\begin{aligned} \varepsilon ^+(\varvec{n}_t) = \max _{0\le \tau \le t}\ \{\varepsilon _0(\varvec{n}_0), \langle \varvec{n}_\tau \cdot \varvec{\varepsilon }_\tau (\varvec{n}_\tau ) \rangle _+\}. \end{aligned}$$

Note that the vectors $\varvec{n}_\tau$ and $\varvec{n}_t$ of the last formula and the previous paragraph must be considered over the initial material undeformed configuration of bone before deformation started. In addition, $\varepsilon _0(\varvec{n}_0)$ is the initial threshold for the occurrence of damage.

#### Proposed constitutive model with damage

Equations (1)–(3), together with (9), describe the mechanical behavior of cortical bone. To compute the partition function, it is necessary to initially determine the number of microstates $g_k = g(E_k)$ that are compatible with the energy $E_k$ of each macrostate. The number $g_k$ gives the number of possible paths for a crack in osteon lattice.

**Fig. 2:**
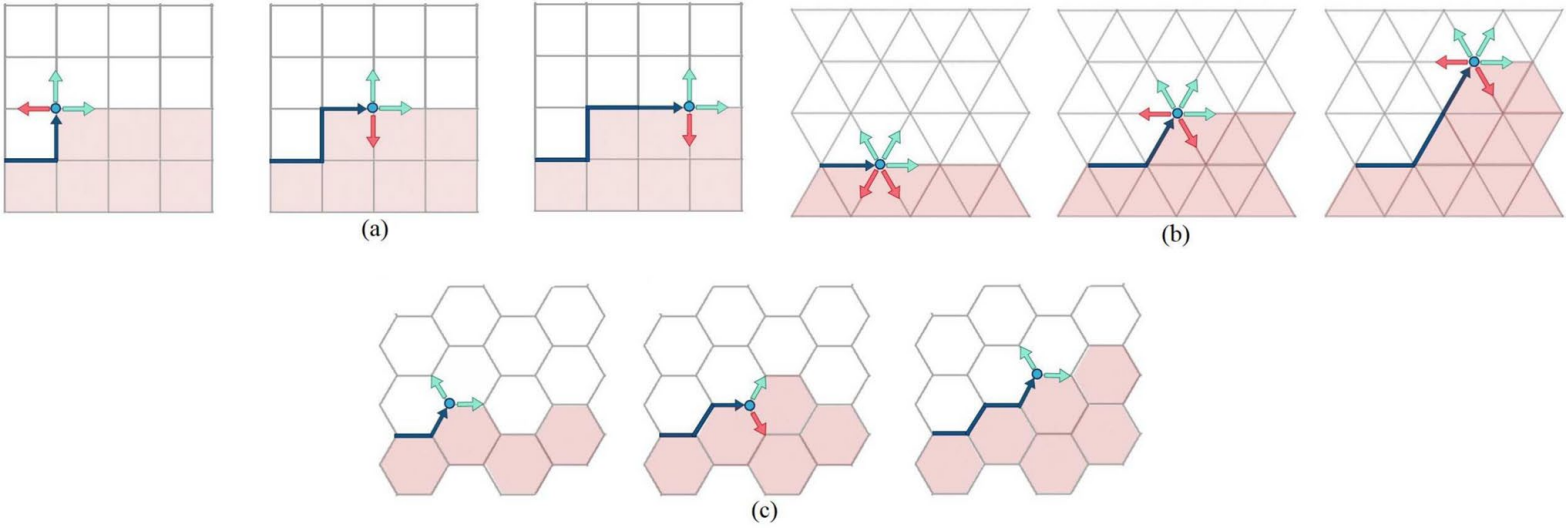
Three idealized osteon configurations (a osteon is located at the center of each cell): **a** *orthogonal lattice*, the crack encounters three possible forward paths $g_k=2^k$, **b** *triangular lattice*, the crack has three permissible directions (without backwards movements) $g_k=3^k$, **c** *hexagonal lattice*, for no backwards movement; there are $2\cdot 1\cdot 2\dots$ possible paths and $g_k \approx \sqrt{2}^{k}$

The computation of the number of possible paths for crack propagation, $g_k=r^k$, where *r* represents the *lattice parameter*, was detailed in our earlier model (García-Vilana and Sánchez-Molina 2023). As illustrated in Fig. 2, this calculation assumes a regular osteonal arrangement, with an osteon at the center of each cell. However, due to the irregular arrangement of osteons in actual specimens, we numerically estimate an average lattice parameter for each sample. Previous analyses suggest that the lattice parameter typically ranges from $1 < r \le 3$.

It is also important to consider that the extent of macrocracking in cortical bone is generally proportional to the number of osteon layers in the cortical thickness. Therefore, the sum in the partition function (1) comprises a finite number, *N*, of terms. Given that the average cortical thickness in human ribs is approximately 0.8 mm and the typical osteon diameter is about 0.2 mm, we anticipate that $N < 5$. This estimation aligns with the numerical values obtained for *N* in our model, as demonstrated by the fittings discussed in “Results”.

Then, the calculation of $g_k$ and the consideration of a finite number of terms lead to the partition function (1) being rewritten as as a closed-form expression

11 $$\begin{aligned} \begin{array}{rl} Z(\alpha ,\varvec{\varepsilon };\beta ,r,N) & = \sum _{k=1}^{N} r^ke^{-\beta E_k(\alpha ,\varvec{\varepsilon })} \\ & = r e^{-\beta (1-\alpha )\psi _0(\varvec{\varepsilon })}\ \frac{1- r^N e^{-\beta N(1-\alpha )\psi _0(\varvec{\varepsilon })} }{1-re^{-\beta (1-\alpha )\psi _0(\varvec{\varepsilon })}}, \end{array} \end{aligned}$$

where the macrostate energies $E_k(\alpha ,\varvec{\varepsilon })=k(1-\alpha )\psi _0(\varvec{\varepsilon })$ are function of three variables: the strain tensor ($\varvec{\varepsilon }$), the damage variable ($\alpha$), and the cracking number (*k*). The additional magnitudes ($\beta ,r,N$) are parameters: $\beta$ is the quenched-disorder parameter, *r* is the lattice parameter, and *N* is the total number of steps of crack advance (which cannot be infinite). In the appendix, the corresponding SDEDF ($\Psi$) is computed. In addition, the constitutive equations are derived using Eq. (3a), resulting in

12 $$\begin{aligned} \varvec{\sigma }(\varvec{\varepsilon }) =\frac{\partial \Psi (\alpha ,\beta ,\varvec{\varepsilon })}{\partial \varvec{\varepsilon }}= -\frac{\partial \ln Z}{\partial \beta }\left( \frac{\partial \ln \psi _0}{\partial \varvec{\varepsilon }} + \frac{\partial \ln (1-\alpha )}{\partial \varvec{\varepsilon }} \right) . \end{aligned}$$

It is demonstrated in the Appendix section “Particularization of stress–strain relationships” that stress can be decomposed into the following three components:

13 $$\begin{aligned} \varvec{\sigma }(\alpha ,\varvec{\varepsilon };\beta ,N,r) = \varvec{\sigma }_0(\varvec{\varepsilon })\cdot \Phi (\alpha ,\varvec{\varepsilon };\beta ,N,r) + \varvec{\sigma }_\alpha (\alpha ,\varvec{\varepsilon };\beta ,N,r), \end{aligned}$$

where $\varvec{\sigma }_0 = \partial \psi _0/\partial \varvec{\varepsilon }$ is the “crackless stress at fixed damage”; representing stress in absence of microcracking and without any variation of damage. In presence of damage and microcracking, the “corrected stress” is $\varvec{\sigma }_0\cdot \Phi$, where the correction factor $\Phi$ is associated with TML, see Eq. (25) in 6.1. Finally, the term $\varvec{\sigma }_\alpha$ is the “increasing-damage stress” and contributes to stress only when $\dot{\alpha }> 0$. In other words, if the cortical bone is subjected to additional loading that exacerbates internal damage, it would generate new internal stresses corresponding to the effort expended in escalating the damage.

### Materials and experimental setting

The experimental data used to calibrate the proposed model were derived from procedures identical to those employed in our previous studies (García-Vilana and Sánchez-Molina 2023; Velázquez-Ameijide et al. 2021). Specifically, these data originated from tensile tests on small cortical bone specimens and bending tests on complete human ribs. We sourced the rib specimens from forensic autopsies conducted at the Forensic Pathology Service of the Legal Medicine and Forensic Science Institute of Catalonia (IMLCFC), ensuring that all specimens came from non-traumatized thorax-related deaths, in accordance with the guidelines set by the monitoring committee of our research center-forensic institute collaboration project.

**Fig. 3:**
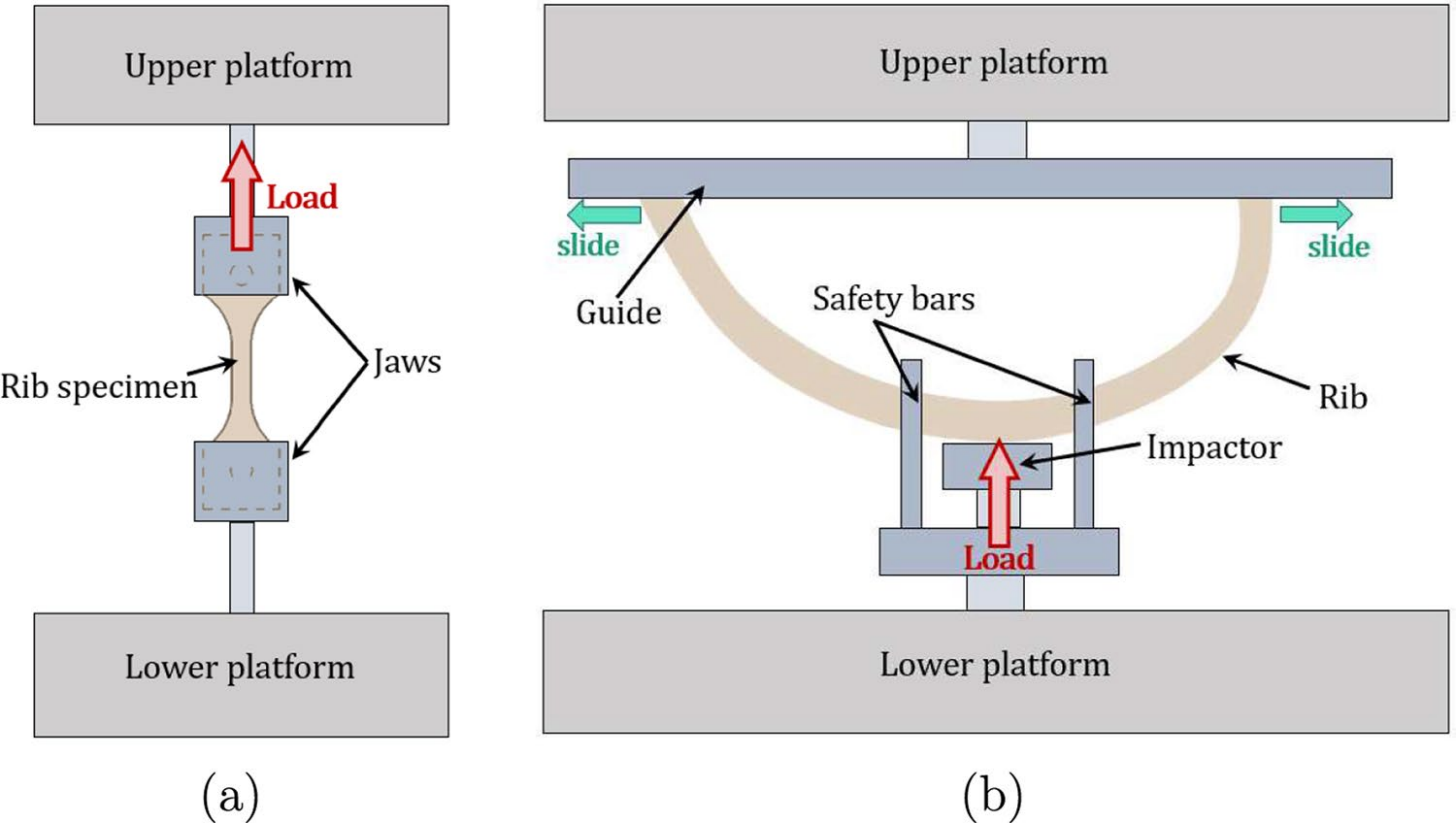
Experimental settings: **a** tensile test for coupon specimens of human rib fixed with special jaws and load longitudinally; **b** three-point bending test of complete rib, where load is applied in the center, while the extremes slide freely inside a guide

For parameter identification, tensile and bending tests were performed using a MicroTEST EM2/20 testing machine, with force measurements obtained via a 500 N HBM S9M load cell connected to a Spider 8-30 acquisition system. The tensile tests involved $n_\textrm{T} = 51$ specimens (35 male and 16 female), aged $55 \pm 20$ years (range 21–91 years). Following the approach in Velázquez-Ameijide et al. (2020), ribs were machined to extract coupons from the anterior section. Soft tissue was first removed, after which cortical slices were cut using a low-speed diamond saw. Two holes, each 2 mm in diameter and spaced 20 mm apart, were drilled into the slices to center and secure them in the clamping system. The slices were then milled into coupons and polished to ensure uniform gauge thickness. The final specimen dimensions were 28 mm in length, 8 mm in width, with a gauge length of 6 mm, a gauge width of 2 mm, and an approximate thickness of 0.5 mm, ensuring a consistent cross-section across the gauge length.

For the bending tests, $n_\textrm{B} = 15$ specimens (10 male and 5 female) were used, aged $55 \pm 11$ years (range 26–62 years). Complete ribs were placed on a sliding guide, with force applied to the outer middle section via a pusher mounted on the lower platform. The rib ends were free to move along the guide, while four aluminum bars installed on the prismatic base stabilized the setup by preventing lateral displacement without contacting the sample. The tensile and bending test setups are illustrated in Fig. 3.

Both tests were conducted under quasi-static conditions, with strain rates maintained below $\dot{\varepsilon } = 0.02\, \text {s}^{-1}$. Digital image correlation (DIC) technology was used to precisely measure displacement and strain fields, enabling accurate stress–strain curves with minimal experimental noise (Velázquez-Ameijide et al. 2021). Detailed methodologies for the tensile and bending tests are provided in our previous works: see Sanchez-Molina et al. (2013), Velázquez-Ameijide et al. (2020) for tensile tests and García-Vilana et al. (2022) for bending tests.

## Results

This section presents the main consequences of the constitutive model (“Consequences of the model”), evaluates the experimental suitability (“Experimental suitability”), and provides values for the model parameters (“Values for the parameters of the model”).

### Consequences of the model

The empirical stress–strain curves start with a nearly constant slope $\partial \sigma /\partial \varepsilon \approx Y_0$ for $\varepsilon \rightarrow 0$. This fact is a necessary consequence of constitutive model, because the strain tensor in the partition function in Eq. (1) contains a sum of exponential functions of a quadratic function, Eq. (6). Then, for intact states ($\alpha \approx 0$), a straightforward computation leads to

14 $$\begin{aligned} \varvec{\sigma } \approx 2 \left[ I_1\left( \mu _2 +\frac{\nu _2}{2} \right) \textbf{I}-\frac{\nu _2}{2}\varvec{\varepsilon } + \rho _2 I_4 \varvec{a}\otimes \varvec{a} + \frac{\tau _2}{2} (\varvec{a}\otimes \varvec{\varepsilon }(\varvec{a})+ \varvec{\varepsilon }(\varvec{a})\otimes \varvec{a}) \right] \underbrace{\frac{\partial \ln Z}{\partial \beta }}_{>0}. \end{aligned}$$

This equation predicts stress–strain curves that exhibit a nearly constant slope, particularly when the strain remains low, because the above relationship approximates a linear function. Our parameter identification from a previous version of the model (referenced in García-Vilana and Sánchez-Molina 2023) confirmed that $\mu _2> -\nu _2/2> 0$, $\rho _2> 0$, and $\tau _2> 0$. These values are critical as they ensure strong ellipticity and pointwise mechanical stability. Specifically, for any non-zero strain tensor, the inequality $\sum _{ijkl} K_{ijkl} E_{ij} E_{kl}> 0$ holds true, where $K_{ijkl} = \partial S_{ij}/\partial E_{kl}$ is the tensor of elastic constants (referenced in Marsden and Hughes (1994)). This condition holds for states with initial damage ($\alpha> 0$) under low strain, Eq. (7) applies. However, in these cases, the elastic constants $K_{ijkl}$ are modified by a factor that reduces stiffness. As a result, the stress–strain curve becomes concave at higher strains, as shown in Fig. 5.

Furthermore, the proposed model is thermodynamically consistent, as damage irreversibly increases. This fact is a consequence of Eq. (32), derived in the appendix. This last equation implies that the derivative of entropy with respect to the damage variable is always positive

15 $$\begin{aligned} \frac{\partial H}{\partial \alpha } = \underbrace{\beta \psi _0(\varvec{\varepsilon })}_{>0} \underbrace{\left[ m_2(k) - m_1^2(k) \right] }_{>0} \underbrace{(1-\alpha )}_{>0}. \end{aligned}$$

In other words, since the sign of changes in entropy and the damage variable are identical, any increase in entropy must correspondingly result in an increase in the damage variable. The first and last terms of the expression (15) are inherently positive by definition. Additionally, the second term can be shown to be positive after calculation using the first and second moments of the cracking number defined in “Partition function and strain-damage energy”:

16 $$\begin{aligned} m_1 = \sum _{\kappa =1}^N \kappa \ p_{\kappa } = \mathbb {E}(k), \qquad m_2 = \sum _{\kappa =1}^N \kappa ^2 p_\kappa = \mathbb {E}(k^2), \end{aligned}$$

where $p_\kappa (\alpha ,\beta ,\varvec{\varepsilon })$ is the probability defined by Eq. (27). Then, the second component enclosed in brackets is precisely the variance of the cracking number $\text {var}(k):= \mathbb {E}(k^2) - \mathbb {E}^2(k)$, which is a positive quantity, by definition.

The fact that damage cannot decrease, as a consequence of the second principle of thermodynamics, is consistently incorporated in the damage evolution equation (9). This is enclosed by the fact that the time derivative of damage variable cannot be negative $\dot{\alpha } \ge 0$, which is consistent with the aforementioned statement.

Another interesting feature of the constitutive model is that it explains why elderly people often show lower yield stress and fracture work, noting these mechanical properties decline with age. The key to this explanation is the stress correction factor, denoted as $\Phi$, which is the term in parentheses in Eq. (13) is influenced by the parameter $\beta$. Crucially, the equation below shows how $\Phi$ decreases as $\beta$ increases

17 $$\begin{aligned} \frac{\partial \Phi }{\partial \beta } = \frac{\partial }{\partial \beta }\left( \frac{1}{1-re^{-\beta \psi _0(1-\alpha )}}-\frac{Nr^Ne^{-\beta N\psi _0(1-\alpha )}}{1-r^Ne^{-\beta N\psi _0(1-\alpha )}}\right) < 0. \end{aligned}$$

**Fig. 4:**
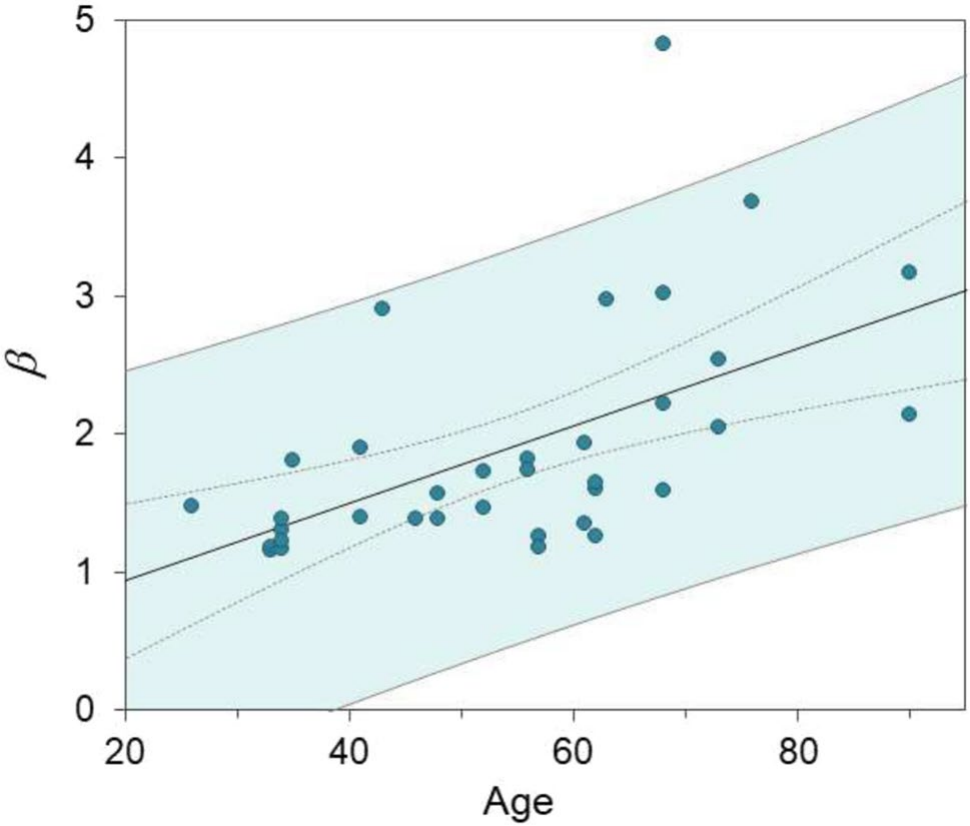
Scatterplot of the *quenched-disorder parameter* ($\beta$), associated with initial microcraking in cortical bone

This indicates that higher values of $\beta$ lead to lower stress levels under constant strain and other conditions. This suggests that the higher $\beta$ values observed in elderly individuals (see Fig. 4) indicate that their bones are more prone to microcracking. This relationship implies that aging increases the quenched-disorder parameter, which is linked to increased microcracking. This microcracking, in turn, contributes to weaker mechanical properties in bones, as evidenced by direct micrographical observations from other researchers, who have demonstrated that an increase in microcracking is responsible for the lower mechanical properties observed, and in weaker bones, the crack path is less deflected (Schwab et al. 2023; Sexton et al. 2024).

### Experimental suitability

**Fig. 5:**
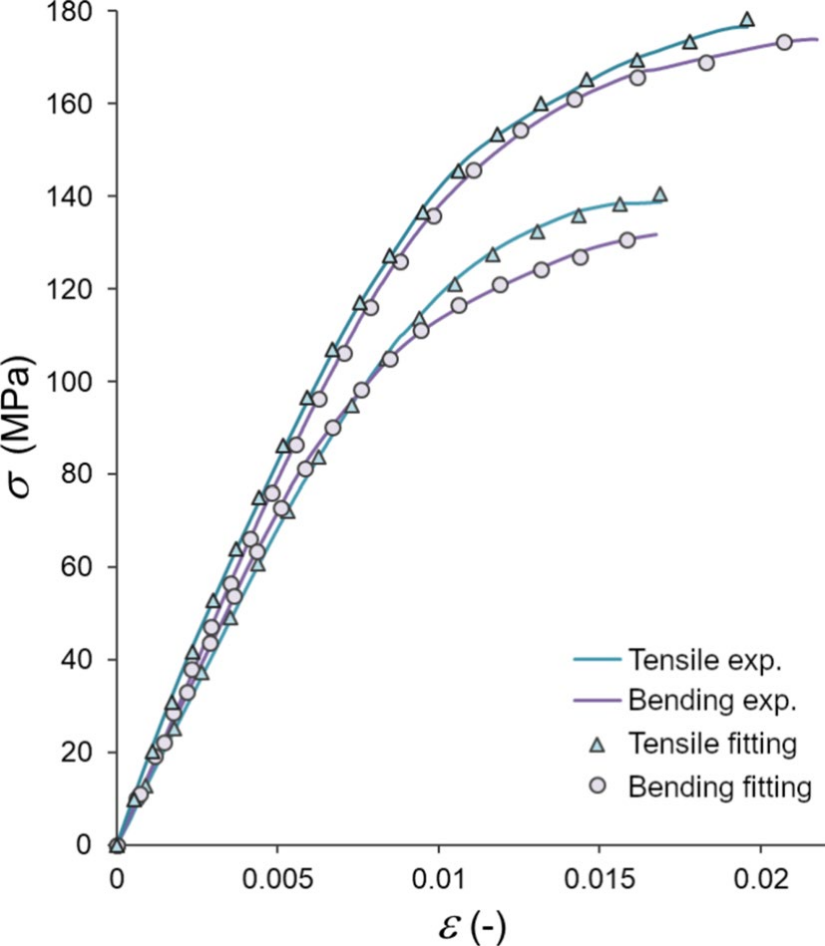
Tensile and bending experimental curves (respct. blue and gray lines) and model fitting (triangles and circles)

**Fig. 6:**
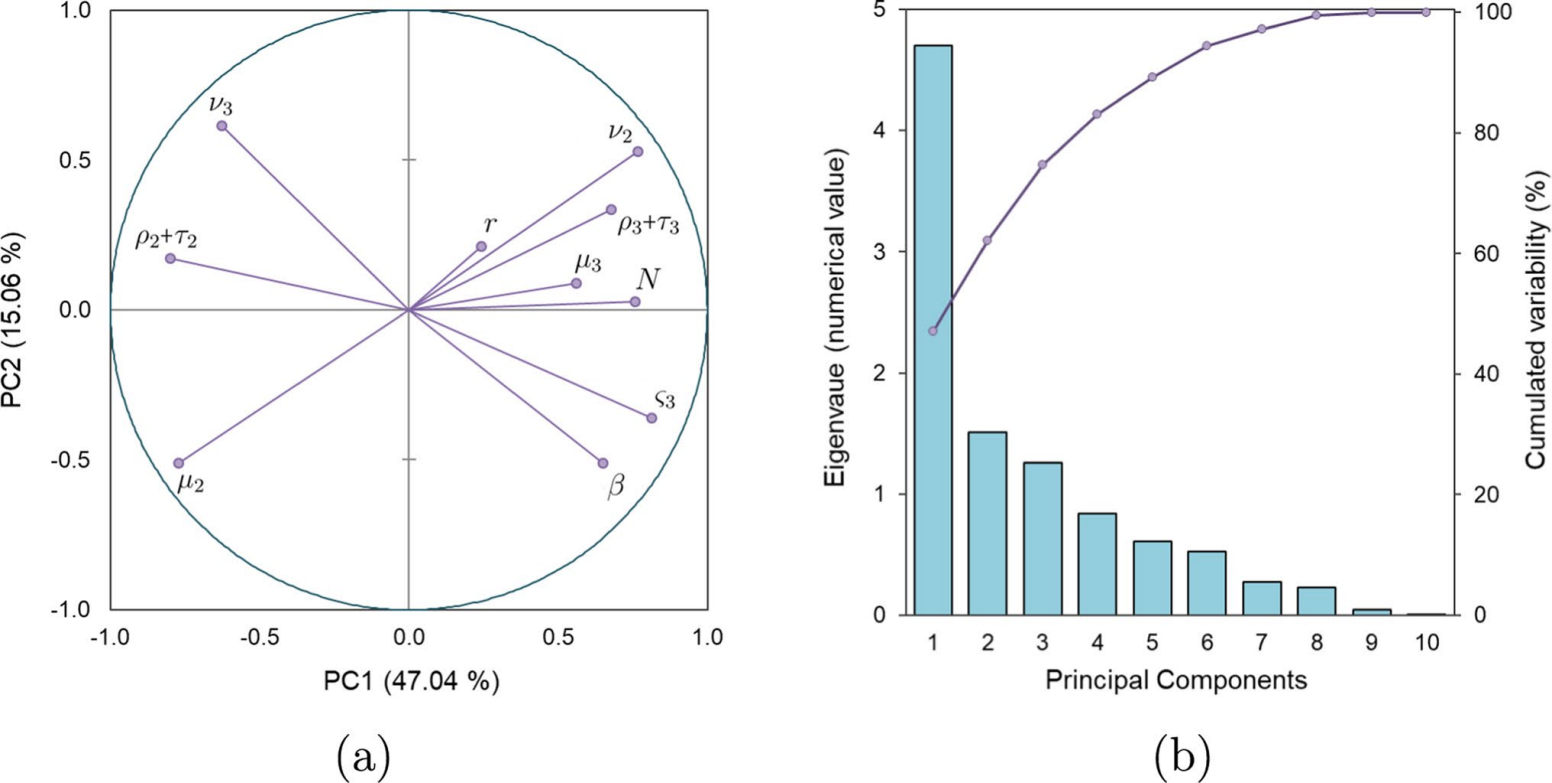
**a** Model parameter correlations with $\hbox {PC}_1$ (horizontal axis) and $\hbox {PC}_2$ (vertical axis) reveal significant dependencies, notably the strong negative correlation ($r = -0.99$) between $\mu _2$ and $\nu _2$. This phenomenon is further discussed in “Particularization of stress–strain relationships”. **b** Sedimentation graph for the weights of the ten principal component analysis (PCA) eigenvalues

To evaluate the suitability of the model, we conducted a comparative analysis between experimental stress–strain curves from mechanical tests and those predicted by the model, through fitting parameters in the constitutive relationships (12) and (13). Figure 5 illustrates four representative stress–strain curves derived from these tests, alongside the model’s predictions. The samples exhibited typical nonlinear responses, notably a reduction in stiffness as strain increased, which the model successfully captured under both tensile and bending scenarios. The model demonstrated remarkable accuracy in replicating the experimental curves, achieving a correlation coefficient ($R^2$) greater than 0.99 for each fit (Fig. 6).

### Values for the parameters of the model

**Fig. 7:**
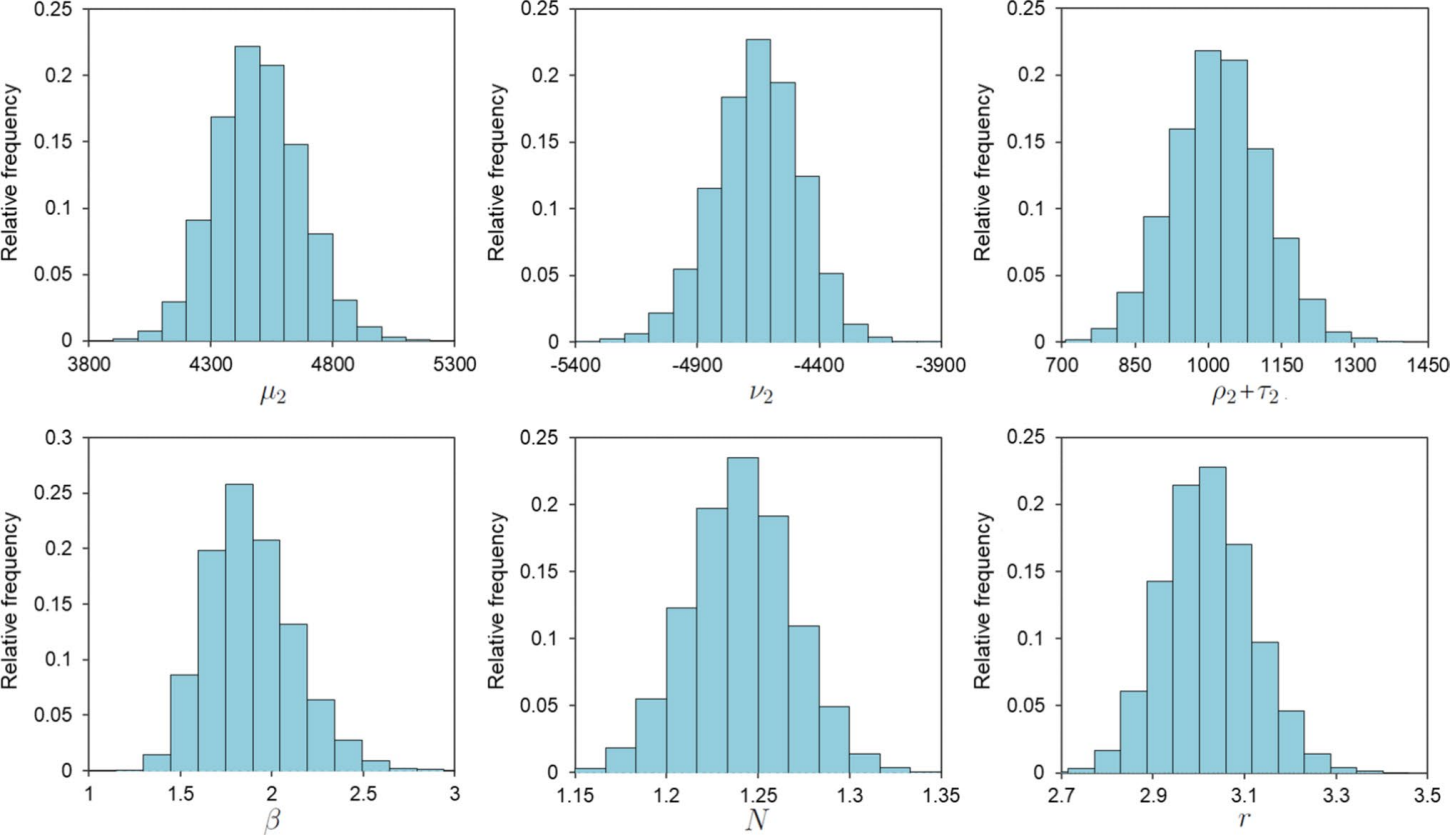
Distributions for the parameters obtained by bootstraping

The model comprises an extensive set of parameters ($\mu _i, \nu _i, \rho _i, \tau _i, \varsigma _i, \beta , r, N$). Under the consistent plane stress conditions of the tests, distinguishing between certain parameters can become unclear. For example, only the combined value of $\rho _i + \tau _i$ can be distinctly measured in tensile tests, rather than their individual contributions. Figure 7 displays the empirical distributions of the main parameters. A correlation analysis among the ten parameters revealed significant interactions, detailed in Table 1 in the appendix. These findings led to the implementation of principal component analysis (PCA), which was used to reduce the dimensionality and mitigate overfitting by collapsing the data into fewer, statistically independent parameters. Remarkably, just three principal components accounted for about 75% of the observed variance in the parameters. Consequently, the parameter vector $\varvec{\theta } = (\mu _i, \nu _i, \rho _i + \tau _i, \varsigma _i, \beta , r, N)$ was effectively simplified to three principal components $\varvec{\lambda } = (\text {PC}_i)_{i=1}^{3}$. These findings are substantiated in Table 2 in “Additional tables concerning the model parameters”, which suggests values for the model parameters based on $\hbox {PC}_1$, $\hbox {PC}_2$, and $\hbox {PC}_3$.

Further analyses also revealed correlations between some parameters and anthropometric variables. Notably, $\hbox {PC}_1$ is positively correlated with age (with a $p-$value of $< 0.007$), whereas $\hbox {PC}_2$ and $\hbox {PC}_3$ show a significant decrease with increasing age (with $p-$values of $< 0.021$ and $< 0.038$, respectively). As a result, the model’s recommended constants are formulated to vary with age, enhancing the model’s applicability across different demographic groups.

## Discussion

This work represents an effective advancement over the previous microcracking model of cortical bone proposed in García-Vilana and Sánchez-Molina (2023). The extended model presented here constitutes a comprehensive constitutive framework that integrates damage mechanics with thermodynamic consistency, grounded in SM principles and supported by a minimal set of assumptions. Notably, it introduces a novel mechanism for microcracking in the inter-osteon space, triggered by pre-existing damage, and formulates an evolution equation for the internal damage variable specific to cortical bone. As in the earlier model, transverse isotropy is assumed due to the predominant longitudinal alignment of osteons in human ribs.

A key feature of this model is its ability to reproduce experimental stress–strain behavior with strong agreement, allowing for the computation of both damage and entropy as internal variables that track the progression of irreversible processes. Importantly, the model now accommodates non-monotonic loading conditions through the introduction of a history-dependent internal damage variable, which encodes information about previously reached overstress levels. In addition, the refined damage evolution equation confirms that entropy consistently increases with damage, in accordance with the second law of thermodynamics.

Rather than aiming to compete with existing heuristic constitutive models for cortical bone, this study seeks to demonstrate the predictive power of SM in explaining macroscopic mechanical behavior from microstructural considerations. To enhance practical applicability, a principal component analysis was conducted to enable individualized parameter estimation based on age, sex, and BMI. The results further suggest that this modeling approach could offer deeper insight into age-related variations in the mechanical properties of bone, grounded in thermodynamic and SM-based reasoning.

Prior research on the nonlinear behavior of cortical bone in axial tensile and more complex situations was validated by the experimental results (Wang et al. 2017; Pezowicz and Glowacki 2012; Velázquez-Ameijide et al. 2020). A quasi-linear elastic behavior is observed under low stress conditions. However, when strain exceeds a certain threshold, there is a noticeable reduction in stiffness (strain softening) and permanent damage occurs. The model predicts that this loss of stiffness is tied to internal microcracking and a non-reducing positive damage variable. Unlike other earlier microcracking models, this proposed model incorporates finite strain, tissue anisotropy, and thermodynamic irreversibility. It also identifies that elderly bones are weaker due to an increased quenched-disorder parameter, which indicates a higher degree of initial microcracking (detailed in “Consequences of the model” and illustrated in Fig. 4). Moreover, the SM approach not only facilitates the numerical computation of entropy but also enhances the accuracy of stress–strain predictions, effectively linking these two phenomena.

Some limitations of the constitutive model described in this work are: (1) Bone remodeling is not accounted for in the model, because it is assumed that stress will increase until fracture occurs (Sansalone et al. 2021; Giorgio et al. 2023), (2) no viscoelastic or strain-rate depending effect has been considered (Sanchez-Molina et al. 2023; Abdel-Wahab et al. 2011; Reisinger et al. 2020; Sánchez-Molina et al. 2020; García-Vilana et al. 2025) (our experimental data consisted only in low-strain rate); therefore, in its current form, the model is not well suited to represent impact or traumatic events involving high strain rates, (3) no viscoplastic effect has been considered in the damage law (Halldin et al. 2014; Lovrenić-Jugović et al. 2020; Reisinger et al. 2024). Aside from the above aspects, another aspect that could be improved is to propose simpler equations for the evolution of the damage variable; see Eqs. (9) and (10). The current form was chosen, because it is the most general rational function, being a homogeneous function of degree one. This provided a natural extension of our previous work (García-Vilana and Sánchez-Molina 2023); however, the obtained values of the parameters suggest that a simpler form could be found by applying some physical principle, or maybe, using some computational efficiency. On the other hand, while this research offers a limited account of the observed variability in the parameters $(\mu _i, \nu _i, \rho _i, \tau _i, \varsigma _i)$ with respect to age-related effects, it does not investigate the influence of physical variables, such as water, mineral, and organic contents on the model parameters (Lee et al. 2021; García-Vilana et al. 2023). Because of the restricted experimental design and absence of precise data, a considerable number of potentially intriguing inquiries remain unresolved in this study. The purpose of the observed variations in the model’s parameters could have been undoubtedly clarified with the addition of densimetric measurements to the mechanical data. Additionally, although the model is intended for non-monotonic loading processes, and Eq. (9) ensures that damage level does not decrease even in non-monotonic processes, these specific behavior was not measured in the experimental conditions.

In addition, this research had a narrow focus on elucidating the consequences of microcracking and damage, thereby neglecting other significant variables, including density distribution (Kerrigan et al. 2014) and local bone mineral density (Helgason et al. 2008; Fonseca et al. 2014; Fleps et al. 2020). Other critical considerations that ought to be incorporated into a more comprehensive model of the cortical bone (Öhman et al. 2011; Ishimoto et al. 2013). In a similar vein, when confronted with non-monotonic loads and situations where the extent of microcracking can be assessed through direct observation utilizing micrographs (Abdel-Wahab et al. 2012; Schwiedrzik et al. 2014), it would be advantageous to apply a generalized version of this model.

## Conclusion

In this work, we have presented a constitutive model for cortical bone that captures the microcracking process using principles from statistical mechanics. The model incorporates finite strain, reflects the material’s transverse isotropy, and describes damage evolution in a thermodynamically consistent manner. This framework naturally accounts for the irreversibility of microcracking and predicts the progressive loss of stiffness once a critical strain threshold is exceeded.

The model was calibrated using experimental data from tensile and bending tests on human rib cortical bone, yielding accurate fits that support its predictive capability. Moreover, we identified meaningful correlations among the model parameters and between these parameters and anthropometric variables. Entropy calculations further reinforce the model’s capacity to track damage progression with increasing strain.

Although the model successfully addresses non-monotonic loading scenarios, it does not yet account for strain-rate-dependent effects, viscoplasticity, or long-term remodeling. Addressing these phenomena will require an extended formulation, which we consider a promising direction for future research.

## Data Availability

Experimental data and regression analyses are available upon request from the authors.

## Appendix

### Computation of the stress–strain relationships

This subsection elucidates the methodologies employed for computing the stress–strain relationships as specified in Eq. (13) and the entropy equation. The foundation for all thermodynamic and constitutive relationships is the strain-dependent energy density function (SDEDF), denoted as $\Psi (\alpha ,\beta ,\varvec{\varepsilon })$, which is a function of the damage variable ($\alpha$), the quenched-disorder parameter ($\beta$), and the strain tensor $(\varvec{\varepsilon })$. The expression for $\Psi (\alpha ,\beta ,\varvec{\varepsilon })$ is derived by substituting the partition function (11) into Eq. (2), resulting in

18 $$\begin{aligned} \Psi = (1-\alpha )\psi _0 -\frac{1}{\beta } \ln \left( r\frac{1-r^N e^{-\beta N(1-\alpha )\psi _0}}{1-re^{-\beta (1-\alpha )\psi _0}} \right) . \end{aligned}$$

For certain computations, it proves beneficial to commence from the summation form of the SDEDF

19 $$\begin{aligned} \Psi = -\frac{\ln Z}{\beta } = -\frac{1}{\beta } \ln \sum _{k=1}^N g_k e^{-\beta k(1-\alpha )\psi _0(\varvec{\varepsilon })}. \end{aligned}$$

This form is advantageous as it requires fewer assumptions and provides greater transparency in the calculations. Notably, it facilitates the derivation of Eq. (12) more straightforwardly from this latter formulation (19) by employing Eq. (3a), which involves obtaining the SDEDF as a derivative with respect to the strain tensor

20 $$\begin{aligned} \varvec{\sigma } = -\frac{1}{\beta Z}\frac{\partial Z}{\partial \varvec{\varepsilon }} = \frac{1}{Z} \frac{\partial [(1-\alpha )\psi _0]}{\partial \varvec{\varepsilon }} \sum _{k=1}^N kg_k e^{-\beta k(1-\alpha )\psi _0(\varvec{\varepsilon })}, \end{aligned}$$

where $\psi _0$ was given in Eq. (4). However, for computational purposes, it is worth calculating of $\ln Z$ with respect to $\beta$ instead of $\varvec{\varepsilon }$

21 $$\begin{aligned} \frac{\partial \ln Z}{\partial \beta } = \frac{1}{Z}\frac{\partial Z}{\partial \beta } = -\frac{(1-\alpha )\psi _0}{Z} \sum _k kg_ke^{-\beta k\psi _0(1-\alpha )}. \end{aligned}$$

Note that (21) differs from (20) in a factor

$$\begin{aligned} -\frac{1}{(1-\alpha )\psi _0}\frac{\partial [(1-\alpha )\psi _0]}{\partial \varvec{\varepsilon }}. \end{aligned}$$

This factor is precisely the derivative of $\ln [(1-\alpha )\psi _0]$ with respect to the strain tensor, which leads us to the following closed-form expression for the stress:

22 $$\begin{aligned} \varvec{\sigma }(\alpha ,\beta ,\varvec{\varepsilon }) = -\frac{1}{\beta }\frac{\partial \ln Z}{\partial \varvec{\varepsilon }} = -\frac{\partial \ln Z}{\partial \beta } \left( \frac{\partial \ln [(1-\alpha )\psi _0]}{\partial \varvec{\varepsilon }} \right) . \end{aligned}$$

This equation is equivalent to Eq. (12) (to see it, expand the logarithm of the product). The previous form easily justifies Eq. (13), which decomposes the stress tensor as $\varvec{\sigma } = \varvec{\sigma }_0\cdot \Phi + \varvec{\sigma }_\alpha$. To simplify the calculations, we rewrite (22) as

23 $$\begin{aligned} \varvec{\sigma }(\alpha ,\beta ,\varvec{\varepsilon }) = \frac{\partial \Psi (\alpha ,\beta ,\varvec{\varepsilon })}{\partial \varvec{\varepsilon }}= -\frac{\partial \ln Z}{\partial \beta }\left( \frac{1}{\psi _0}\frac{\partial \psi _0}{\partial \varvec{\varepsilon }} - \frac{1}{1-\alpha } \frac{\partial \alpha }{\partial \varvec{\varepsilon }} \right) . \end{aligned}$$

And then, we calculate the two derivatives in the last parenthesis. The first derivative of the last parenthesis gives rise to the $\varvec{\sigma }_0$ part of the stress tensor

24 $$\begin{aligned} \frac{\partial \psi _0}{\partial \varvec{\varepsilon }} = 2 \left[ I_1 \left( \mu _2 +\frac{\nu _2}{2}\right) \textbf{I}-\frac{\nu _2}{2}\varvec{\varepsilon } + \rho _2 I_4\varvec{a}\otimes \varvec{a} + \frac{\tau _2}{2} ( \varvec{a}\otimes \varvec{\varepsilon }(\varvec{a})+ \varvec{\varepsilon }(\varvec{a})\otimes \varvec{a} ) \right] =: \varvec{\sigma }_0. \end{aligned}$$

Now, the final summation common to formulas (20) and (21), which as will be seen is related to the TML, will define the correction factor $\Phi$, i.e., the pre-term that multiplies the previous derivative within (23)

25 $$\begin{aligned} \Phi (\alpha ,\varvec{\varepsilon };\beta ,N,r):= -\frac{\partial \ln Z}{\partial \beta }\frac{1}{\psi _0}. \end{aligned}$$

This term matches exactly with the expected value of the cracking number calculated in Eq. (28) and, therefore, is a measure of the progression of cracking or TML. For situations where the damage variable is not increasing ($\dot{\alpha } = 0$), the stress is given by combining Eqs. (24) and (25), that is, $\varvec{\sigma } = \varvec{\sigma }_0\cdot \Phi$. Finally, for situations where damage is occurring ($\dot{\alpha }> 0$), the damage evolves according to Eq. (9) and, therefore, we have

26 $$\begin{aligned} \varvec{\sigma }_\alpha := +\frac{\partial \ln Z}{\partial \beta }\left( \frac{1}{1-\alpha } \frac{\text {d}\tilde{\alpha }}{\text {d}\varvec{\varepsilon }} \right) , \end{aligned}$$

where the last derivative can be calculated from Eq. (8).

### Entropy computation

Before proceeding to the entropy calculation, we write the probabilities of the Maxwell–Boltzmann (M–B) distribution for the different macrostates associated with the partition function (1). For a canonical ensemble with a macrostate characterized by the state variables ($\alpha , \beta , \varvec{\varepsilon }$), the probability of having a cracking number *k* is given by

27 $$\begin{aligned} p_k = \frac{g_k}{Z} e^{-k\beta \hat{E}(1-\alpha )} = \frac{g_k\ e^{-\beta \hat{E}(1-\alpha )}}{\sum _{k=1}^N g_k\ e^{-k \beta \hat{E} (1-\alpha )}}, \end{aligned}$$

where $\hat{E}:= \psi _0(\varvec{\varepsilon })$. The first moment for the M–B distribution, the expected value of the cracking number (*k*), is therefore

28 $$\begin{aligned} \mathbb {E}(k) = \sum _{\kappa =1}^N \kappa \ p_\kappa = \frac{\sum _{\kappa =1}^N g_\kappa \kappa \ e^{-\kappa \beta \hat{E} (1-\alpha )}}{\sum _{\kappa =1}^N g_\kappa \ e^{-\kappa \beta \hat{E} (1-\alpha )}} = m_1(k). \end{aligned}$$

And the expected value of TML $(k\ell _0)$ would therefore be $\mathbb {E}(\text {TML}) = \ell _0 \mathbb {E}(k)$, which also coincides with the factor $\Phi$ from Eq. (25). The second moment of the distribution, which will also be used later, is given by

29 $$\begin{aligned} \mathbb {E}(k^2) = \sum _{\kappa =1}^N \kappa ^2\ p_\kappa = \frac{\sum _{\kappa =1}^N g_\kappa \kappa ^2\ e^{-\kappa \beta \hat{E} (1-\alpha )}}{\sum _{\kappa =1}^N g_\kappa \ e^{-\kappa \beta \hat{E} (1-\alpha )}} = m_2(k). \end{aligned}$$

The expressions (28) and (29) are involved in the entropy calculation. The entropy is given by Eq. (3b), and developing the formula and introducing the partition function in the form of a summation, we obtain

30 $$\begin{aligned} H= \ln Z -\beta \frac{\partial \ln Z}{\partial \beta } = \ln Z + \beta \hat{E}(1-\alpha ) \frac{\sum _{\kappa =1}^N g_\kappa \kappa \ e^{-\kappa \beta \hat{E} (1-\alpha )}}{\sum _{\kappa =1}^N g_\kappa \ e^{-\kappa \beta \hat{E} (1-\alpha )}}, \end{aligned}$$

where the last term is precisely the $m_1(k)$ from Eq. (28) and, therefore, the equation can be expressed in terms of TML

31 $$\begin{aligned} H = \ln Z + \beta \hat{E} \frac{1-\alpha }{\ell _0} \mathbb {E}(\text {TML}). \end{aligned}$$

If we now derive the expression (30) for entropy with respect to the damage variable ($\alpha$), to obtain (15), we arrive at

32 $$\begin{aligned} \frac{\partial H}{\partial \alpha } = (\beta \psi _0)^2\left[ \frac{\sum _{\kappa =1}^N g_\kappa \kappa ^2\ e^{-\kappa \beta \hat{E} (1-\alpha )}}{\sum _{\kappa =1}^N g_\kappa \ e^{-\kappa \beta \hat{E} (1-\alpha )}} - \left( \frac{\sum _{\kappa =1}^N g_\kappa \kappa \ e^{-\kappa \beta \hat{E} (1-\alpha )}}{\sum _{\kappa =1}^N g_\kappa \ e^{-\kappa \beta \hat{E} (1-\alpha )}} \right) ^2 \right] (1-\alpha ). \end{aligned}$$

Note that, compared to Eqs. (28) and (29), the term in brackets is the variance $\text {var}(k) = m_2(k)-m_1^2(k)> 0$. This is precisely Eq. (15) of “Consequences of the model”.

### Particularization of stress–strain relationships

In the conducted mechanical tests, most of the stress and strain components are null. Particularly, the stress and strain tensors take the following special form

33 $$\begin{aligned} \varvec{\sigma } = \begin{bmatrix} \sigma & \tau & 0 \\ \tau & 0 & 0 \\ 0 & 0 & 0 \end{bmatrix}, \qquad \qquad \varvec{\varepsilon } = \begin{bmatrix} \varepsilon _l & \gamma & 0 \\ \gamma & \varepsilon _t & 0 \\ 0 & 0 & \varepsilon _t \end{bmatrix}, \end{aligned}$$

where $\varepsilon _l$ is the strain in the longitudinal direction of the rib, $\varepsilon _t$ is the strain in the transverse directions, and $\gamma$ is the angular distortion. Now, the stress–strain components are explicitly written in terms of these three components: $\varepsilon _l, \varepsilon _t, \gamma$. First, the components of the “uncorrected basic stress” ($\varvec{\sigma }_0$) were obtained previously. The result of introducing the special strain tensor (33), in relationships (24b) are the following linear functions:

34a $$\begin{aligned}&\begin{array}{rl} \sigma ^{(0)}_{xx} = (\varepsilon _l+2\varepsilon _t)(2\mu _2+\nu _2) - \nu _2\varepsilon _l + 2(\rho _2+\tau _2)\varepsilon _l \end{array} \end{aligned}$$

34b $$\begin{aligned}&\begin{array}{rl} \sigma ^{(0)}_{zz} = \sigma ^{(0)}_{yy} = (\varepsilon _l+2\varepsilon _t)(2\mu _2+\nu _2) - \nu _2 \varepsilon _t \end{array} \end{aligned}$$

34c $$\begin{aligned}&\begin{array}{rl} \sigma ^{(0)}_{xy} = (\tau _2 - \nu _2) \gamma . \end{array} \end{aligned}$$

It is important to note that the first and second equations suggest that, for bone for low strain and no damage, the Poisson effect can be expressed as

35 $$\begin{aligned} \nu _{lt} = -\frac{\varepsilon _t}{\varepsilon _l} \approx \frac{2\mu _2+\nu _2}{4\mu _2+\nu _2} \approx 0.325. \end{aligned}$$

However, this aligns with the findings of certain scholars (Reilly and Burstein 1975; Abdi et al. 2024). In fact, this correlation between $\mu _2$ and $\nu _2$ explains why the obtained values for all samples are correlated.

Finally, the computation of the components of $\varvec{\sigma }_\alpha$ leads to

36a $$\begin{aligned}&\sigma ^{(\alpha )}_{xx} = -\frac{1}{1-\alpha } \left( \frac{\partial \ln Z}{\partial \beta }\right) \left[ \frac{(2\bar{\nu }-1)^2[(4\bar{\nu }+1)\mu _3 - 2\bar{\nu } \nu _3]}{(2\bar{\nu }-1)^2\mu _2+\bar{\nu }(2\bar{\nu }-1)\nu _2 + \rho _2+\tau _2 } +\dots \right. \nonumber \\&\quad \left. \dots + \frac{\rho _2+\tau _2 - \bar{\nu } \varsigma _3 }{(2\bar{\nu }-1)^2\mu _2+\bar{\nu }(2\bar{\nu }-1)\nu _2 + \rho _2+\tau _2} \right] \end{aligned}$$

36b $$\begin{aligned}&\quad \sigma ^{(\alpha )}_{yy} = \sigma ^{(\alpha )}_{zz} = \frac{1}{1-\alpha } \left( \frac{\partial \ln Z}{\partial \beta }\right) \left[ \mu _2\frac{(2\bar{\nu }-1)^4\mu _3 +(2\bar{\nu }-1)^3\nu _3 - (4\bar{\nu }-2)(\tau _3+\rho _3)-2\varsigma _3}{[(2\bar{\nu }-1)^2\mu _2+\bar{\nu }(2\bar{\nu }-1)\nu _2 + \rho _2+\tau _2]^2} - \dots \right. \nonumber \\&\quad \dots - \nu _2\frac{(2\bar{\nu }-1)^3(2\bar{\nu }+1)\mu _3+\bar{\nu }^2(2\bar{\nu }-1)^2\nu _3-(1+\bar{\nu })(\rho _3+\tau _3)+\bar{\nu }^3\varsigma _3 }{[(2\bar{\nu }-1)^2\mu _2+\bar{\nu }(2\bar{\nu }-1)\nu _2 + \rho _2+\tau _2]^2} - \dots \end{aligned}$$

36c $$\begin{aligned}&\quad \left. \dots - (\rho _2+\tau _2)\frac{3(2\bar{\nu }-1)^2\mu _3 +(2\bar{\nu }-1)^2\nu _3 - \bar{\nu }\varsigma _3}{[(2\bar{\nu }-1)^2\mu _2+\bar{\nu }(2\bar{\nu }-1)\nu _2 + \rho _2+\tau _2]^2} \right] \nonumber \\&\quad \sigma ^{(\alpha )}_{xy} = -4\frac{1}{1-\alpha } \left( \frac{\partial \ln Z}{\partial \beta }\right) \frac{\nu _2(\rho _3+\tau _3+\mu _3)-\nu _3(\rho _2+\tau _2+\mu _2)}{(\mu _2+\rho _2+\tau _2)^2} \frac{\gamma }{\varepsilon _l}, \end{aligned}$$

where we have used for brevity $\bar{\nu }:= -\varepsilon _l/\varepsilon _t$. All the above equations were used in fitting using least squares.

### Additional tables concerning the model parameters

In this section, additional quantitative information is provided regarding the value of the calculated parameters. For the entire sample of 66 individuals ($n_\textrm{T} = 51$, from tensile test specimens and $n_\textrm{B} = 15$ from bending test specimens), each parameter mentioned in “Values for the parameters of the model” was calculated. As explained in the same section, the mechanical parameters of the model are not independent of each other, but there are some significant correlations between them as shown in Table 1.

**Table 1:** Spearman correlation coefficients

Variables	$\mu _2$	$\nu _2$	$\rho _2+\tau _2$	$\mu _3$	$\nu _3$	$\rho _3+\tau _3$	$\varsigma _3$	$\beta$	N	r
$\mu _2$	–	$-$ 0.990*	0.421*	$-$ 0.349*	0.260	$-$ 0.604*	$-$ 0.399*	$-$ 0.317*	$-$ 0.577*	$-$ 0.119
$\nu _2$		–	$-$ 0.414*	0.358*	$-$ 0.249	0.607*	0.405*	0.289	0.558*	0.135
$\rho _2+\tau _2$			–	$-$ 0.495*	0.473*	$-$ 0.475*	$-$ 0.678*	$-$ 0.492*	$-$ 0.661*	$-$ 0.216
$\mu _3$				–	$-$ 0.052	0.229	0.688*	0.187	0.270	0.244
$\nu _3$					–	$-$ 0.247	$-$ 0.694*	$-$ 0.691*	$-$ 0.431*	$-$ 0.042
$\rho _3+\tau _3$						–	0.325*	0.378*	0.453*	0.236
$\varsigma _3$							–	0.576*	0.503*	0.258
$\beta$								–	0.395*	$-$ 0.056
N									–	0.049

Significant correlations (*p*-value $<0.05$) are marked with an asterisk $(*)$

To facilitate standardized values of parameters, usable in computational models, the value of each parameter is expressed as

37 $$\begin{aligned} \pi _i = \bar{\pi }_i + \pi _{i,1} \text {PC}_1 + \pi _{i,2} \text {PC}_2 + \pi _{i,3} \text {PC}_3, \end{aligned}$$

where the numerical values of $\bar{\pi }_i$ and $\pi _{i,j}$ can be easily obtained from Table 2. When no additional information is available, the recommendation is to set $\text {PC}_1 = \text {PC}_2 = \text {PC}_3 = 0$, and thus, a reasonable approximation for each parameter, $\bar{\pi }_i$, results as a result of this choice. If additional information, such as age, sex (sex = 0 for females and 1 for males), or body mass index (BMI), is known, then the following equations allow to estimate the principal components $\hbox {PC}_i$:

38 $$\begin{aligned} {\left\{ \begin{array}{ll} \text {PC}_1 = -4.621 + 0.055\ \text {age} - 0.338\ \text {sex} + 0.063\ \text {BMI} \\ \text {PC}_2 = 1.696 -0.027\ \text {age} + 0.812\ \text {sex} -0.810\ \text {BMI} \\ \text {PC}_3 = -1.332 - 0.015\ \text {age} +0.158\ \text {sex} + 0.080\ \text {BMI}. \end{array}\right. } \end{aligned}$$

Then, a better approximation for the mechanical parameters results. Using prior estimations in Eq. (37) yields a result for the parameter values that is more closely tailored to the individual’s characteristics.

**Table 2:** Values for the parameters of the model

Parameter	Equation	$r^2$
$\mu _2$	$= -361.50\ \text {PC}_1 -369.68\ \text {PC}_2 +265.95\ \text {PC}_3 + 4497.2$	0.735
$\nu _2$	$= +373.06\ \text {PC}_1 +386.14\ \text {PC}_2 -253.62\ \text {PC}_3 -4654.8$	0.740
$\rho _2+\tau _2$	$= -217.06\ \text {PC}_1 + 53.88\ \text {PC}_2 -85.77\ \text {PC}_3 + 1021.4$	0.631
$\mu _3$	$= +32465\ \text {PC}_1 + 5465\ \text {PC}_2 + 61093\ \text {PC}_3 - 302633$	0.587
$\nu _3$	$= -30177\ \text {PC}_1 + 53603\ \text {PC}_2 + 26813\text {PC}_3 -144006$	0.731
$\rho _3+\tau _3$	$= +7168\ \text {PC}_1 + 8694\ \text {PC}_2 -3171\ \text {PC}_3 -24863$	0.541
$\varsigma _3$	$= +63552\ \text {PC}_1 -45127\ \text {PC}_2 +42299\ \text {PC}_3 -194644$	0.737
$\beta$	$= +0,2197\ \text {PC}_1 -0,3617\ \text {PC}_2 -0.0961\ \text {PC}_3 1,7507$	0.537
*r*	$= +0.0191\ \text {PC}_1 +0.0281\ \text {PC}_2 +0.1065\ \text {PC}_3 +1.2406$	0.516
*N*	$= +0.1948\ \text {PC}_1 +0.0549 \text {PC}_2 -0,1779\ \text {PC}_3 + 3.0201$	0.568

## Abbreviations

DIC: Digital image correlation
PMHS: *Post mortem* human subject
SDEDF: Strain-damage energy density function
SEDF: Strain energy density function
SM: Statistical mechanics
TML: Total microcracking length
